# Five-year risk of fracture and subsequent fractures among adults with cerebral palsy

**DOI:** 10.1016/j.bonr.2022.101613

**Published:** 2022-08-20

**Authors:** Daniel G. Whitney, Gregory A. Clines, Aleda M. Leis, Michelle S. Caird, Edward A. Hurvitz

**Affiliations:** a Department of Physical Medicine and Rehabilitation, University of Michigan, Ann Arbor, MI, USA; b Institute for Healthcare Policy and Innovation, University of Michigan, Ann Arbor, MI, USA; c Division of Metabolism, Endocrinology & Diabetes, Department of Internal Medicine, University of Michigan, Ann Arbor, MI, USA; d Endocrinology Section, Ann Arbor VA Medical Center, Ann Arbor, MI, USA; e Epidemiology Department, University of Michigan, Ann Arbor, MI, USA; f Department of Orthopaedic Surgery, University of Michigan, Ann Arbor, MI, USA

**Keywords:** CP, cerebral palsy, ICD, International Classification of Diseases, Ninth or Tenth Revision, Clinical Modification, CI, confidence interval, IR, incidence rate, IRR, incidence rate ratio, Cerebral palsy, Fracture, Prevention, Subsequent fracture

## Abstract

**Background:**

Epidemiologic evidence documenting the incidence of fracture and subsequent fractures among adults with cerebral palsy (CP) is lacking, which could inform fracture prevention efforts. The objective was to characterize the 5-year rate of initial and subsequent fragility fractures among adults with CP.

**Methods:**

This retrospective cohort study used Medicare claims from 01/01/2008–12/31/2019 from adults ≥18 years old with CP (*n* = 44,239) and elderly ≥65 years old without CP (*n* = 2,176,463) as a comparison. The incidence rate (IR), IR ratio (IRR), and site distribution were estimated for the initial and subsequent fragility fractures over 5-years by sex and age.

**Results:**

The IR of fragility fracture at any site over the 5-year follow-up was similar for 18–30-year-old men with CP (IR = 5.2; 95%CI = 4.4–5.9) and 30–34-year-old women with CP (IR = 6.3; 95%CI = 5.3–7.2) compared to the same sex youngest-old (65–74 years old) without CP (IRR = 1.09 and 0.94, respectively, both *P* > 0.05), and increased with older age for those with CP. The number of fragility fractures and IR of subsequent fragility fractures was similar for young men and middle-aged women with CP compared to elderly without CP, and increased with older age for those with CP. The proportion of fragility fracture at the tibia/fibula decreased while the vertebral column and multiple simultaneous sites (most involved hip/lower extremities) increased with older age.

**Conclusion:**

Young and middle-aged adults with CP had similar-to-worse initial and subsequent fragility fracture profiles compared to the general elderly population- a well characterized group for bone fragility. Findings emphasize the need for fracture prevention efforts at younger ages for CP, possibly by ~5 decades younger.

## Highlights

• Fracture prevention efforts are focused on the elderly and postmenopausal women.
• Fragility fracture risk is elevated for those with CP across the adult lifespan.
• For men, the risk for 18–29 year olds (CP) was on par with 65–74 year olds (no CP).
• For women, risk for 30–34 year olds (CP) was on par with 65–74 year olds (no CP).
• CP had a higher risk of subsequent fragility fractures that was also premature.

## 1 Introduction

Bone fragility is a lifelong problem for individuals with cerebral palsy (CP). Due to a variety of interacting factors, children with CP can develop small and structurally fragile bones (Whitney et al., 2017; Henderson et al., 2010; Henderson et al., 2005; Modlesky et al., 2009). Abnormal bone development increases fracture risk of the lower extremities in the pediatric years (Whitney et al., 2021a) and predisposes to accelerated bone strength declines throughout the adult years. For example, the prevalence of fracture at any site is up to 6.5 times higher for adults with vs. without CP of the same age and sex strata (Whitney et al., 2021a). Moreover, population-based studies have reported strong associations between fragility fractures with incident cardiorespiratory morbidity and all-cause mortality among adults with CP, and that the fracture-associated disease sequela was present for women and men with CP and prior to reaching their elderly years (Etter et al., 2020; Whitney et al., 2020a; Whitney et al., 2020b; Whitney et al., 2022a).

Collectively, the growing body of evidence suggests that fragility fractures represent a major and costly (Whitney et al., 2022b) burden for individuals with CP across the adult life course and not just in the elderly years. Yet, policy, public health, and clinical efforts for fracture prevention are often focused on elderly and postmenopausal women at risk for fragility fractures (Force et al., 2018; Burge et al., 2007), which is too late to begin fracture prevention efforts for many individuals with CP. To date, there is little epidemiologic evidence on the incidence of fragility fractures across the adult life course for individuals CP. Such knowledge may inform population-based fracture prevention efforts, including at what stage of adulthood to implement prevention strategies given the unique skeletal needs of adults with CP (Whitney et al., 2021a).

Additionally, there is little epidemiologic evidence on subsequent fragility fracture risk (i.e., following the initial fracture) among adults with CP. Studies in non-CP elderly cohorts have shown that the risk of subsequent fractures can reach up to 42 % within 5-years of the initial fracture, which further increases mortality rate and healthcare costs beyond the effect of a single fracture (Balasubramanian et al., 2019; Bliuc et al., 2009; Weaver et al., 2017). Importantly, the extent of these post-fracture effects varied based on the location of the initial and/or subsequent fracture site (Balasubramanian et al., 2019; Alarkawi et al., 2020). Anti-resorption medication exposure is associated with reduced fragility fracture risk among adults with CP, but this association exhibited effects by fracture site (Whitney et al., 2021b). Therefore, characterizing subsequent fragility fracture risk profiles among adults with CP can help inform post-fracture healthcare monitoring and treatment strategies to mitigate burdens following an initial fracture.

The objectives of this study were to describe the initial (primary objective) and subsequent (secondary objective) fragility fracture characteristics (e.g., rate, site, number of fractures) over 5 years of follow-up among women and men ≥18 years old with CP. To enhance policy and clinical interpretations, an elderly (≥65 years old) cohort without CP was included as a comparison. The rationale is that deriving estimates compared to elderly without CP, a well-characterized clinical group for bone fragility, may better position the evidence to determine if a greater emphasis on CP and younger age should be considered for changes in fracture prevention related policy and clinical protocols.

## 2 Material and methods

### 2.1 Data source

This retrospective cohort study used administrative claims data from 01/01/2008–12/31/2019 from the Medicare fee-for-service database, which has representation across the U.S. This database is vast, so researchers often obtain access to a random 5 % or 20 % sample of the unique beneficiaries (along with all their claims) that provides reasonable representation of all beneficiaries. This study obtained access to a 20 % random sample. This database has four parts that captures the type of insurance coverage for the beneficiary. This study obtained access to two parts, Part A (hospital insurance) and B (medical insurance), that provides the necessary information needed to conduct this study. This study did not have access to Part C (Medicare Advantage plan) or D (prescription drug coverage). Medicare is a federal program that provides health insurance to the elderly, as well as elderly and non-elderly with specific disabilities (including CP) or with end-stage kidney disease. Medicare beneficiaries can have dual eligibility with Medicaid, but Medicare pays first for services that are also covered by Medicaid, including the diagnostic services needed to conduct this study. For research studies, clinical conditions (e.g., CP, fracture) are identified by searching for unique codes attached to claims that are primarily used for billing reimbursement. Since the data are de-identified prior to administering to researchers, patient consent was not required and the University's Institutional Review Board approved this study as non-regulated.

### 2.2 Cohort selection and follow-up time

**Fig. 1:**
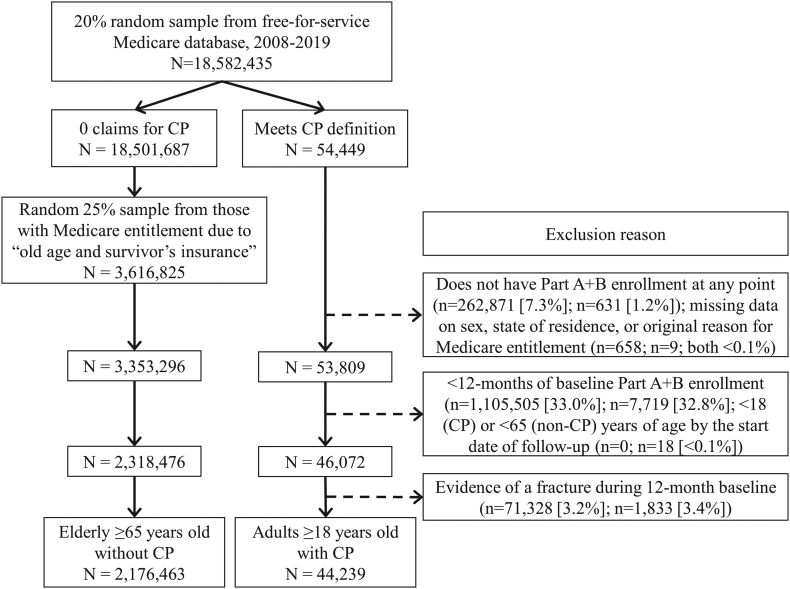
Flow chart of inclusion/exclusion criteria to derive the analytic samples of adults with cerebral palsy (CP) and elderly without CP.

A flow chart to derive the analytic cohorts is presented in Fig. 1. The start date of follow-up (i.e., time 0) was 1-year after the individual's earliest enrollment date between 01/01/2008–12/31/2013 to obtain a 1-year baseline period and allow for up to 5-years of follow-up after time 0 (through 12/31/2019). Adults with CP who were ≥ 18 years old by their start date, with continuous enrollment in Part A and B during the 1-year baseline period and for ≥1 day of follow-up, without a fracture in the 1-year baseline period, and without missing data on sex were eligible for analysis. Adults with CP were identified by ≥1 inpatient claim or ≥ 2 outpatient claims containing a pertinent International Classification of Diseases Clinical Modification code for CP (ICD-9 codes: 333.71, 343.0–343.4, 343.8, 343.9; ICD-10 codes: G80.x), where the outpatient claims were on separate days within 12-months of one another.

Elderly individuals ≥65 years old without CP were included if their original reason for Medicare entitlement was due to “old age and survivor's insurance” to exclude individuals with other non-CP disabilities in this age range. Given the large volume of eligible participants, a random 25 % sample was first derived for computing purposes, then all other inclusion/exclusion criteria used for CP were applied (Fig. 1).

This study allowed up to 3 gaps in Part A and B enrollment during the follow-up period, where each gap lasted ≤1-month. The goal of this approach was to optimize sufficient follow-up information to mitigate bias from truncated follow-up periods. The majority of the final cohorts had no gaps in enrollment during the follow-up (≥96.8 %), while only ≤0.1 % had 3 gaps. Sensitivity analysis found that the study findings were unchanged when including those without any gaps (data not shown).

### 2.3 Initial and subsequent fragility fractures

A fragility fracture was defined as a fracture without a trauma code (e.g., motor vehicle accident) ±7 days from the fracture date (Keshishian et al., 2017), while all other fractures were considered to be “trauma” fractures. The first fragility fracture after time 0 was identified at the following sites: vertebral column; hip including proximal femur; non-proximal femur; tibia/fibula; humerus; ulna/radius; multiple simultaneous sites; or at an unspecified site.

To capture distinct subsequent fragility fracture events, a fragility fracture at a different site from the preceding fracture was included. If the subsequent fragility fracture was at the same site as the preceding fracture, a gap of ≥6-months from the last claim date was required, which is longer than what has been used in previous claims-based studies (Keshishian et al., 2017; Liu et al., 2018). Fracture healing and recovery may take longer for adults with vs. without CP, requiring a more conservative approach to capture distinct subsequent fragility fractures. This process was repeated up to 2 times for a total of 3 fragility fractures during the follow-up (1 initial and up to 2 subsequent fragility fractures), as the number of subsequent fractures after 3 was too small for analysis.

### 2.4 Patient-level characteristics

Age, sex, race, region of residence in the U.S., and the original reason for Medicare entitlement were retrieved. As individuals could move locations during the follow-up period, U.S. region of residence was described based on the single reported region if it had not changed or the number of moves during the individual's study period (baseline + follow-up).

### 2.5 Statistical analysis

Baseline descriptive characteristics were summarized for the cohorts. The remainder of the analyses were stratified by sex and age categories. Age was categorized for the primary objective as 3 young groups (18–29, 30–34, 35–40 years), 5 middle-aged groups (41–44, 45–49, 50–54, 55–59, 60–64 years), and 3 conventional elderly groups (Alterovitz and Mendelsohn, 2013), including the youngest-old (65–74 years), middle-old (75–84 years), and oldest-old (≥85 years). For the secondary objective, age was categorized more broadly as young (18–40 years), middle-aged (41–64 years), and elderly (≥65 years).

For the primary objective, the incidence rate (IR with 95 % confidence intervals [CI]) of the first fragility fracture at any site was estimated as the number of events divided by person-time multiplied by 100. Individuals were followed from time 0 to the fracture or other censor event (trauma fracture, death, loss of continuous enrollment), whichever came first. Person-time for this analysis was calculated as the total person-days divided by 1826.25 to estimate over a 5-year period. The IR ratio (IRR) was estimated for non-elderly with CP by comparing each young and middle-aged age group to the youngest-old without CP, while the IRR was estimated for elderly adults with vs. without CP within the same age category.

For the secondary objective, the number of fragility fractures over the follow-up period was described as 0, 1, 2, or ≥ 3 fragility fractures. The number of fragility fractures for adults with CP was compared to elderly without CP for each sex separately using an unadjusted generalized linear model with zero-inflated Poisson distribution, which is a two-part model that is useful for dealing with count data that has excess zero counts (Lambert, 1992). The first part models the probability of having 0 fragility fractures vs. ≥1 fragility fracture, while the second part models the count of fragility fractures conditional on having ≥1 fragility fracture. The log of follow-up time was included as the offset to account for the different follow-up time for each person.

For those with an initial fragility fracture, the time-to-fracture and IR were estimated for the 2nd fragility fracture after re-setting time 0 to the initial fracture date. The same process was repeated for the 3rd fragility fracture among those with 2 fragility fractures. Thus, the IR for the subsequent fragility fractures represents the time from the preceding fragility fracture. For these analyses, person-time was calculated as the total person-days divided by 365.25 to estimate over a 1-year period. The site distribution of fragility fractures was described as the proportion of those that sustained a fragility fracture, and compared between adults with CP and elderly without CP using the Chi-squared test.

### 2.6 Sensitivity analysis

A sensitivity analysis was conducted to assess for possible bias by period effects on the primary outcome (initial fragility fracture), as the standard of care may have changed over this time period. Specifically, a descriptive analysis was performed that documented the frequency and proportion of the initial fracture that occurred per year during the follow-up based on the study entry year. As most individuals were already enrolled in Medicare prior to 2008, the focus of the interpretation was on the relative proportion of any fragility fracture over the 5-year follow-up.

Estimates that had <11 cases were not reported or were suppressed to comply with the Data Use Agreement for patient de-identification purposes. Analyses were performed using Statistical Analysis Software version 9.4 and *P* < 0.05 (two-tailed) was considered statistically significant.

## 3 Results

There were 44,239 adults ≥18 years old with CP and 2.2 million elderly ≥65 years old without CP eligible for analysis. Baseline descriptive characteristics of the cohorts are presented in Table 1.

**Table 1:** Baseline characteristics of adults with cerebral palsy (CP) and elderly without CP.

	CP (*n* = 44,239)	Elderly without CP (*n* = 2,176,463)
Age, mean (SD)	48.3 (15.7)	73.5 (7.8)
18–40 years, % (n)	34.0 (15,023)	0 (0)
41–64 years, % (n)	48.9 (21,649)	0 (0)
≥ 65 years, % (n)	17.1 (7567)	100.0 (2,176,463)
Sex, % (n)		
Female	46.6 (20,624)	57.2 (1,244,760)
Male	53.4 (23,615)	42.8 (931,703)
Race, % (n)		
Asian	1.0 (421)	2.0 (44,194)
Black	13.1 (5796)	7.9 (171,133)
Hispanic	3.1 (1380)	1.9 (41,844)
North American Native	0.8 (355)	0.3 (6822)
White	80.1 (35,417)	85.1 (1,851,444)
Other	2.0 (870)	2.8 (61,026)
U.S. region of residence, % (n)		
Midwest	24.4 (10,800)	21.3 (462,969)
Northeast	19.7 (8713)	17.4 (378,433)
South	32.1 (14,208)	33.0 (717,477)
West	16.1 (7106)	19.2 (417,770)
Unknown	0.3 (109)	1.0 (22,421)
Moved, 2 locations	6.7 (2956)	7.6 (165,310)
Moved, ≥3 locations	0.8 (347)	0.6 (12,083)
Original reason for Medicare entitlement, % (n)		
Old age and survivor's insurance	8.6 (3814)	100.0 (2,176,463)
Disability insurance benefits (DIB)	91.0 (40,238)	0 (0)
End-stage renal disease (ESRD)	0.2 (90)	0 (0)
Both DIB and ESRD	0.2 (97)	0 (0)

SD, standard deviation.

### 3.1 Primary objective

**Fig. 2:**
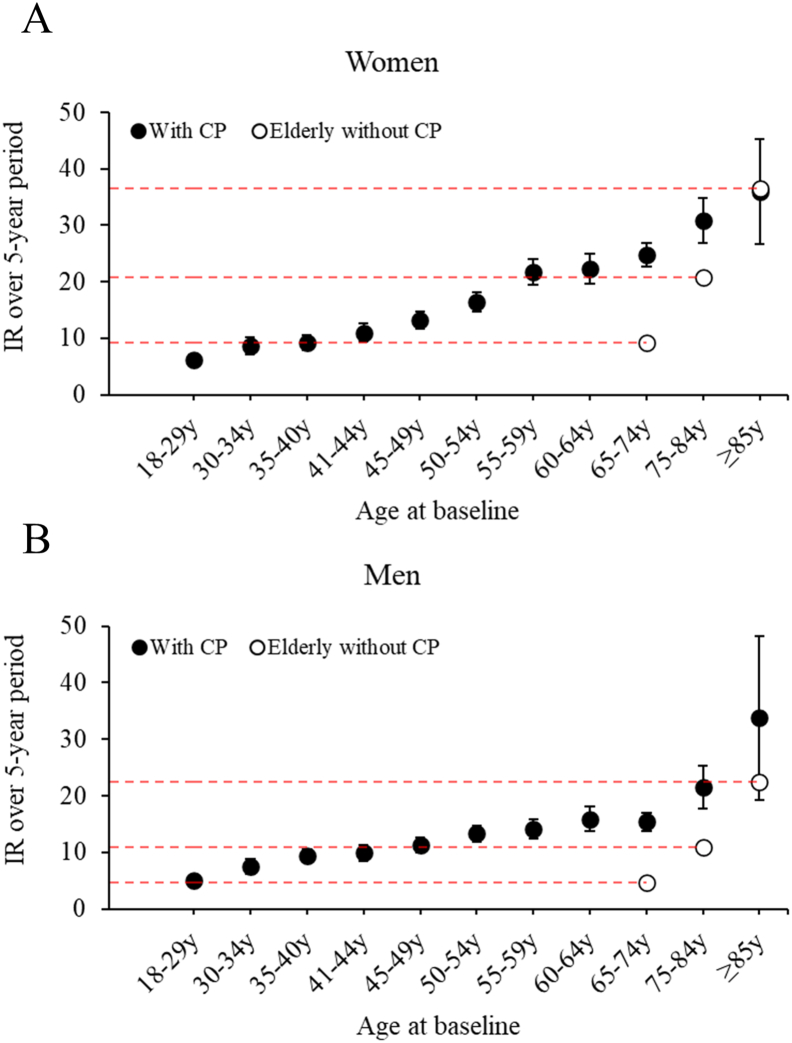
Incidence rate (IR) of fragility fracture at any site over a 5-year follow-up for (A) women with cerebral palsy (CP) vs. elderly women without CP and (B) men with CP vs. elderly men without CP by age group. The circle represents the IR estimate and the vertical lines represent the 95 % confidence interval. The 95 % confidence intervals are very small for elderly women and men without CP and difficult to visualize. The horizontal dashed red lines represent the IR for the 3 elderly age groups to ease visual comparison for the CP age groups.

The IR of the first fragility fracture at any site over the 5-year follow-up is presented in Table 2 and visually in Fig. 2. The IR increased with older age for women and men with and without CP.

**Table 2:** Incidence rate (IR) of the first fragility fracture (any site) over the 5-year follow-up and censor reason for adults with cerebral palsy (CP) and elderly without CP stratified by sex and age group in years (y).

	Censor reason if not end of follow-up			Incidence of fragility fracture		
	Trauma fracture % (n)	Death % (n)	Loss to follow-up % (n)	Fragility fracture event % (n)	IR over 5-year period (95 % CI)	IRR (95 % CI)
Women						
With CP						
18–29y	1.1 (31)	2.9 (85)	6.3 (183)	5.8 (169)	6.3 (5.3, 7.2)	0.68 (0.59, 0.79)^1^
30–34y	1.2 (19)	3.4 (54)	4.4 (69)	7.9 (124)	8.6 (7.1, 10.2)	0.94 (0.79, 1.12)^1^
35–40y	1.1 (25)	3.2 (73)	5.1 (118)	8.4 (193)	9.2 (7.9, 10.5)	1.01 (0.87, 1.16)^1^
41–44y	1.5 (28)	4.1 (77)	4.4 (82)	9.9 (185)	11.0 (9.4, 12.6)	1.20 (1.04, 1.39)^1^
45–49y	2.3 (55)	5.0 (122)	3.5 (85)	11.7 (285)	13.2 (11.6, 14.7)	1.44 (1.28, 1.61)^1^
50–54y	1.7 (38)	4.9 (110)	4.1 (91)	14.3 (322)	16.4 (14.6, 18.2)	1.79 (1.60, 2.00)^1^
55–59y	1.9 (36)	7.2 (136)	3.8 (72)	18.1 (342)	21.8 (19.5, 24.1)	2.37 (2.13, 2.64)^1^
60–64y	2.5 (37)	8.3 (121)	4.5 (65)	18.5 (270)	22.4 (19.7, 25.1)	2.44 (2.17, 2.75)^1^
65–74y	2.9 (76)	9.1 (242)	5.0 (133)	20.1 (531)	24.7 (22.6, 26.8)	2.70 (2.48, 2.94)^1^
75–84y	2.7 (28)	19.1 (198)	6.9 (72)	22.4 (232)	30.8 (26.8, 34.8)	1.48 (1.30, 1.68)^2^
≥85y	*	31.5 (79)	*	23.1 (58)	36.0 (26.8, 45.3)	0.99 (0.76, 1.28)^3^
Without CP						
65–74y	0.9 (6667)	4.6 (34,958)	4.8 (36,585)	8.3 (63,275)	9.2 (9.1, 9.2)	Reference^1^
75–84y	2.2 (7281)	13.4 (44,731)	5.9 (19,701)	16.7 (55,592)	20.9 (20.7, 21.0)	Reference^2^
≥85y	3.3 (4896)	32.5 (48,892)	9.2 (13,851)	22.3 (33,492)	36.5 (36.2, 36.9)	Reference^3^
Men						
With CP						
18–29y	1.1 (38)	3.8 (136)	4.6 (165)	4.8 (174)	5.2 (4.4, 5.9)	1.09 (0.94, 1.27)^1^
30–34y	1.0 (18)	4.1 (74)	6.0 (110)	6.8 (125)	7.5 (6.2, 8.8)	1.58 (1.33, 1.88)^1^
35–40y	1.4 (39)	4.0 (111)	5.1 (142)	8.6 (238)	9.4 (8.2, 10.6)	1.99 (1.75, 2.27)^1^
41–44y	1.5 (34)	4.8 (106)	4.9 (108)	9.0 (199)	9.9 (8.6, 11.3)	2.10 (1.83, 2.42)^1^
45–49y	1.2 (34)	5.1 (148)	3.7 (109)	10.1 (297)	11.3 (10.0, 12.6)	2.38 (2.13, 2.67)^1^
50–54y	1.8 (50)	6.8 (189)	4.5 (124)	11.7 (324)	13.3 (11.9, 14.8)	2.81 (2.52, 3.14)^1^
55–59y	1.3 (29)	8.6 (192)	4.1 (92)	12.3 (276)	14.2 (12.5, 15.9)	3.00 (2.67, 3.38)^1^
60–64y	2.1 (33)	10.6 (169)	4.1 (66)	13.5 (215)	15.9 (13.8, 18.0)	3.36 (2.94, 3.85)^1^
65–74y	1.7 (46)	11.8 (321)	4.3 (117)	13.0 (352)	15.4 (13.8, 17.0)	3.25 (2.93, 3.61)^1^
75–84y	*	26.0 (210)	*	15.7 (127)	21.6 (17.8, 25.3)	1.96 (1.65, 2.34)^2^
≥85y	*	37.6 (41)	*	19.3 (21)	33.7 (19.3, 48.2)	1.50 (0.98, 2.30)^3^
Without CP						
65–74y	0.5 (3342)	7.3 (45,466)	4.4 (27,569)	4.3 (27,157)	4.7 (4.7, 4.8)	Reference^1^
75–84y	1.1 (2651)	19.3 (44,880)	6.4 (14,981)	9.0 (20,842)	11.0 (10.8, 11.1)	Reference^2^
≥85y	2.0 (1450)	39.7 (28,637)	11.2 (8057)	13.9 (10,050)	22.6 (22.1, 23.0)	Reference^3^

CI, confidence interval; IRR, IR ratio. *N < 11 in at least one of the cells in the row, resulting in data suppression for patient de-identification purposes. ^1-3^Corresponds to the reference cohort to estimate the IRR.

For women, the IR of the first fragility fracture at any site was similar for 30–34 year olds with CP as compared to the youngest-old (65–74 years old) without CP. The IRR among non-elderly women with CP compared to the youngest-old women without CP ranged from 32 % lower (18–30 years old) to 144 % higher (60–64 years old). The IRR comparing women with vs. without CP was 170 % higher for the youngest-old, 48 % higher for the middle-old, and similar for the oldest-old due to the rapidly increasing IR with age group from elderly women without CP.

For men, the IR of the first fragility fracture at any site was similar for 18–30 year olds with CP as compared to the youngest-old without CP. The IRR among non-elderly men with CP compared to the youngest-old men without CP ranged from 9 % higher (18–30 years old) to 236 % higher (60–64 years old). The IRR was higher for elderly men with vs. without CP.

The 3 most common sites of the first fragility fracture among women with CP was the tibia/fibula (34.3 % to 15.8 %, decreasing with age), multiple sites involving the lower extremities (24.5 % to 43.7 %, increasing with age), and vertebral column (9.7 % to 18.1 %, increasing with age) (data not shown due to small sample sizes for sites by the narrowly defined age groups). A similar pattern was observed for men with CP. The distribution of fracture site for elderly women and men with CP was similar to elderly women and men without CP.

### 3.2 Secondary objective

**Fig. 3:**
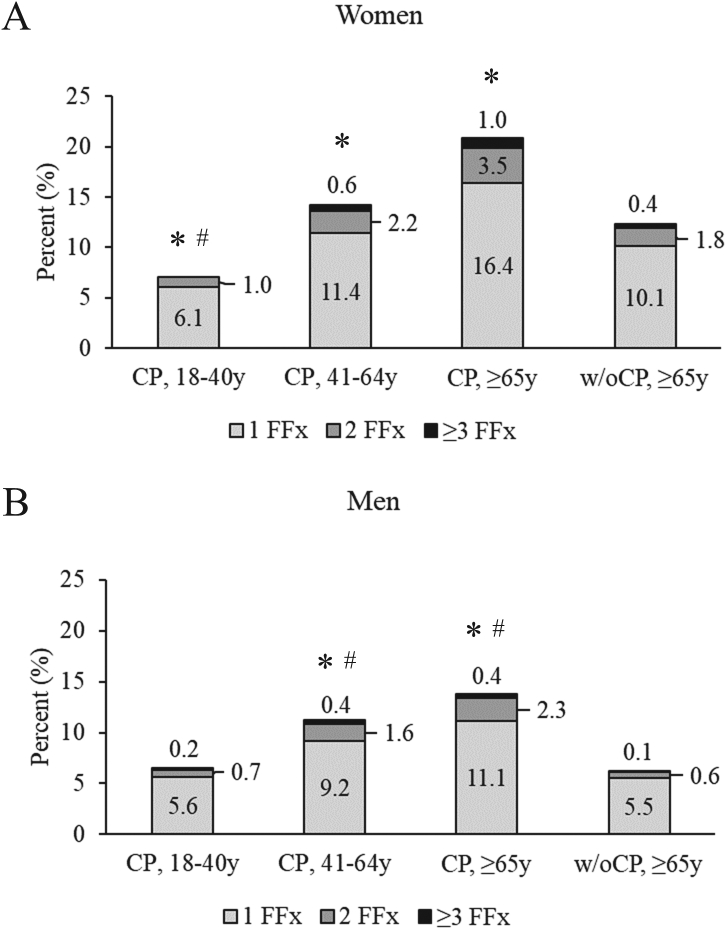
The proportion of adults with cerebral palsy (CP) by young (18–40 years [y]), middle-aged (41-64y), and elderly (≥65y) groups and elderly without CP (w/oCP) with 1 to ≥3 fragility fractures (FFx) over the 5-year follow-up for (A) women and (B) men. For panel A, the 2 and ≥ 3 FFx categories are combined for young women with CP for patient de-identification purposes (*n* < 11 for one of the categories). *P < 0.05 compared to elderly w/oCP based on the zero-inflated part of the generalized linear model with a zero-inflated Poisson distribution. #*P* < 0.05 compared to elderly w/oCP based on the count part of the generalized linear model with a zero-inflated Poisson distribution.

The proportion that sustained ≥1 fragility fracture over the follow-up increased with age and was higher for middle-aged and elderly women and men with CP as compared to elderly without CP (all *P* < 0.05) (Fig. 3). Among those that sustained ≥1 fragility fracture, the number of total fragility fractures sustained over the follow-up was lower for young women with CP and higher for middle-aged and elderly men with CP as compared to the same sex elderly without CP (all *P* < 0.05).

The time-to-fracture decreased and the IR increased with each subsequent fragility fracture for all groups, except young women with CP (Table 3). For women, the IR of subsequent fragility fractures was lower for young women with CP and similar for middle-aged and elderly women with CP as compared to elderly women without CP. For men, the IR of subsequent fragility fractures was similar for young men with CP as compared to elderly men without CP. For middle-aged and elderly men with CP, the IR of the 2nd fragility fracture was higher, but the IR of the 3rd fragility fracture was similar as compared to elderly men without CP.

**Table 3:** Incidence rate (IR) and IR ratio (IRR) of the first and subsequent fragility fractures (any site) and time to fracture* during the 5-year follow-up for adults with cerebral palsy (CP) and elderly without CP (w/oCP) stratified by sex and age group in years (y).

	CP, 18-39y	CP, 40-64y	CP, ≥65y	w/oCP, ≥65y
Women				
1st fracture for entire cohort				
Sample size (n)	6795	9894	3935	1,244,760
Time (days) to fracture, median (IQR)	808 (386, 1337)	793 (372, 1293)	769 (355, 1248)	788 (363, 1272)
IR per 100 person years (95 % CI)	**1.6 (1.4, 1.7)**	**3.3 (3.1, 3.4)**	**5.4 (5.0, 5.7)**	2.9 (2.9, 2.9)
IRR (95 % CI), ref.: w/oCP, ≥65y	**0.54 (0.49, 0.59)**	**1.12 (1.07, 1.19)**	**1.85 (1.72, 1.98)**	Reference
2nd fracture for those with 1st fracture				
Sample size (n)	486	1404	821	152,359
Time (days) to fracture, median (IQR)	514 (397, 834)	549 (357, 786)	527 (361, 768)	544 (363, 834)
IR per 100 person years (95 % CI)	**6.4 (4.9, 7.9)**	9.1 (8.0, 10.2)	10.6 (9.1, 12.2)	9.3 (9.2, 9.4)
IRR (95 % CI), ref.: w/oCP, ≥65y	**0.69 (0.54, 0.87)**	0.98 (0.87, 1.10)	1.14 (0.98, 1.32)	Reference
3rd fracture for those with 2 prior fractures				
Sample size (n)	69	276	175	27,152
Time (days) to fracture, median (IQR)	647 (420, 827)	457 (291, 633)	435 (315, 636)	426 (308, 616)
IR per 100 person years (95 % CI)	**5.9 (1.5, 10.2)**	15.0 (11.1, 18.8)	15.5 (10.6, 20.4)	15.0 (14.6, 15.5)
IRR (95 % CI), ref.: w/oCP, ≥65y	**0.39 (0.19, 0.82)**	1.00 (0.77, 1.29)	1.03 (0.75, 1.41)	Reference

Men				
1st fracture for entire cohort				
Sample size (n)	8228	11,755	3632	931,703
Time (days) to fracture, median (IQR)	856 (407, 1335)	852 (432, 1317)	778 (349, 1264)	828 (394, 1315)
IR per 100 person years (95 % CI)	1.4 (1.3, 1.5)	**2.5 (2.4, 2.7)**	**3.4 (3.1, 3.7)**	1.4 (1.4, 1.4)
IRR (95 % CI), ref.: w/oCP, ≥65y	0.99 (0.91, 1.08)	**1.76 (1.67, 1.86)**	**2.37 (2.17, 2.59)**	Reference
2nd fracture for those with 1st fracture				
Sample size (n)	537	1311	500	58,049
Time (days) to fracture, median (IQR)	421 (287, 791)	488 (341, 814)	607 (367, 915)	512 (350, 789)
IR per 100 person years (95 % CI)	6.4 (4.9, 7.8)	**8.5 (7.4, 9.6)**	**9.7 (7.8, 11.7)**	7.0 (6.8, 7.1)
IRR (95 % CI), ref.: w/oCP, ≥65y	0.91 (0.73, 1.15)	**1.21 (1.07, 1.39)**	**1.40 (1.14, 1.70)**	Reference
3rd fracture for those with 2 prior fractures				
Sample size (n)	74	231	97	7003
Time (days) to fracture, median (IQR)	402 (312, 506)	435 (274, 581)	504 (219, 685)	416 (293, 591)
IR per 100 person years (95 % CI)	12.3 (5.3, 19.2)	14.9 (10.8, 19.1)	13.4 (6.4, 20.4)	12.9 (12.1, 13.7)
IRR (95 % CI), ref.: w/oCP, ≥65y	0.95 (0.54, 1.68)	1.16 (0.87, 1.54)	1.04 (0.61, 1.76)	Reference

IQR, interquartile range; CI, confidence interval. *The time was assessed for those that sustained a fragility fracture. Statistically significant differences at *P* < 0.05 for the IR and IRR of any fracture compared to elderly adults without CP (w/oCP, ≥65y) is bolded for ease of interpretation.

**Fig. 4:**
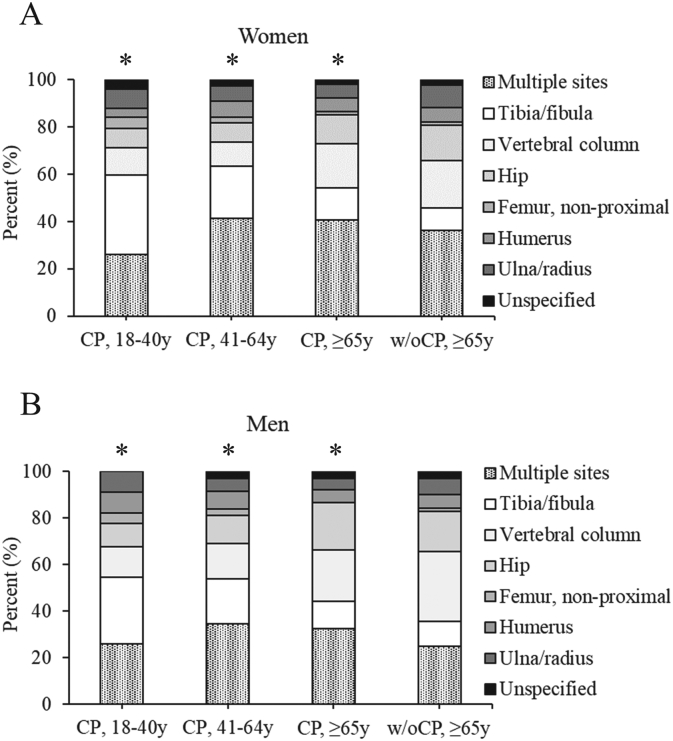
The site distribution of the first fragility fracture for adults with cerebral palsy (CP) by young (18–40 years [y]), middle-aged (41-64y), and elderly (≥65y) groups and elderly without CP (w/oCP) over the 5-year follow-up for (A) women and (B) men. For panel B, the ulna/radius and unspecified categories were combined for young men with CP and the hip and femur categories were combined for elderly men with CP for patient de-identification purposes (*n* < 11 for one of the categories). *P ≤ 0.001 compared to elderly w/oCP using the Chi-squared test.

The site distribution of the first fragility fracture was different for women and men with CP as compared to elderly without CP (all *P* < 0.001) (Fig. 4). The site distribution of subsequent fragility fractures is not displayed in tables or figures as some of the age and sex stratified fracture sites had *n* < 11 cases, but the predominant site was multiple simultaneous sites for subsequent fragility fractures (majority involved the lower extremities).

### 3.3 Sensitivity analysis

The majority of the cohort with CP (85.0 %) and without CP (83.1 %) had a study entry year in 2009, and similar study entry years thereafter (with CP, 2.5 % to 3.7 %; without CP, 2.9 % to 3.9 %). The frequency and proportion of the initial fragility fracture for the entire cohort with CP and elderly without CP is presented in Table 4. As most individuals were enrolled prior to the study period, the majority of fragility fractures occurred in the cohorts with a start date in 2009. However, there was no strong evidence that fragility fracture patterns over the follow-up differed considerably within cohorts based on the study entry year.

**Table 4:** Sensitivity analysis showing the frequency and proportion of the initial fragility fracture per year of follow-up based on the study entry year for adults ≥18 years old with cerebral palsy (CP; *n* = 5509) and elderly ≥65 without CP (*n* = 210,408).

Study entry year	Follow-up year 1	Follow-up year 2	Follow-up year 3	Follow-up year 4	Follow-up year 5
	% (n)	% (n)	% (n)	% (n)	% (n)
With CP					
2009	24.0 (1033)	21.3 (917)	19.2 (828)	18.2 (783)	17.2 (741)
2010	39.2 (49)	21.6 (27)	14.4 (18)	14.4 (18)	10.4 (13)
2011	32.4 (47)	17.9 (26)	14.5 (21)	25.5 (37)	9.7 (14)
2012	31.2 (58)	20.4 (38)	22.0 (41)	18.8 (35)	*
2013	28.7 (41)	21.7 (31)	18.9 (27)	17.5 (25)	13.3 (19)
2014	36.1 (57)	22.8 (36)	15.2 (24)	20.3 (32)	*
Without CP					
2009	25.2 (44,030)	21.6 (37,852)	19.3 (33,739)	17.5 (30,687)	16.3 (28,560)
2010	32.2 (1952)	19.5 (1180)	19.8 (1198)	19.3 (1170)	9.2 (560)
2011	30.6 (1857)	19.8 (1199)	21.0 (1275)	19.9 (1205)	8.6 (523)
2012	29.8 (2384)	19.9 (1588)	20.0 (1601)	19.9 (1588)	10.4 (832)
2013	31.8 (2284)	19.9 (1428)	19.2 (1381)	19.3 (1387)	9.7 (697)
2014	34.6 (2852)	19.3 (1592)	18.9 (1562)	18.7 (1545)	8.5 (700)

*N < 11 in at least one of the cells in the row, resulting in data suppression for patient de-identification purposes.

## 4 Discussion

One main finding from this large, nationwide study is that the 5-year rate of a fragility fracture at any site among 18–30 year old men with CP and 30–34 year old women with CP was on par with the youngest-old (65–74 years old) without CP, and increased with older age for CP. Although, it is important to note the high fracture rate for 18–29 year old women with CP that was slightly less than the youngest-old women without CP. Considering that 65 years old is the age at which most medical organizations recommend screening (e.g., dual-energy X-ray absorptiometry [DXA] scans) in women and regarded as an early at-risk age to bolster fracture prevention efforts, these findings suggest that the early at-risk window may begin 40–50 years earlier for adults with CP. Another main finding is that the number and rate of subsequent fragility fractures among young men (18–40 years old) with CP and middle-aged women (41–64 years old) with CP was similar to elderly (≥65 years old) without CP, and increased with older age for CP. Prior studies have found that adults with vs. without CP have higher post-fracture morbidity and mortality burdens even prior to reaching their elderly years (Etter et al., 2020; Whitney et al., 2020a; Whitney et al., 2020b; Whitney et al., 2022a), and that subsequent fractures can exacerbate costly health declines in elderly without CP (Balasubramanian et al., 2019; Bliuc et al., 2009; Weaver et al., 2017). Taken together, widespread policy, public health, and clinical efforts are needed to improve fracture prevention at much younger ages for adults with CP as compared to the general population.

Study findings from the elderly cohorts without CP are consistent with prior studies, suggesting usability of Medicare claims to derive estimates for adults with CP. For example, large prospective cohort studies from Australia and Norway reported the IR of an initial fracture to be 2.6 to 3.2 per 100 person-years for women and 0.9 to 1.6 per 100 person-years for men ≥50 or ≥ 60 years old (Alarkawi et al., 2020; Center et al., 2007), which is similar to the IR of 2.9 for women and 1.4 for men ≥65 years old observed in the current study.

In the current study, adults with CP had a different site distribution of fragility fractures compared to elderly without CP. For adults with CP, the proportion of tibia/fibula fragility fractures started high and decreased with older age, while the proportion of vertebral column fragility fractures increased with older age. The proportion of fragility fractures at multiple simultaneous sites also increased with older age, with the majority involving the hip and/or lower extremities. Interpretations are limited as to whether the proportion that experienced fragility fractures at the tibia/fibula was truly decreasing, as it may have been encompassed within the multiple simultaneous site variable. Further, approximately 3 in 4 vertebral column fractures in older women are not detected clinically at the time of the fracture (Fink et al., 2005). It is unknown if there is differential sensitivity in clinically identifying vertebral fractures in people with vs. without CP and possible effects by age and sex. As this study only had an elderly group without CP for comparison, it is unknown if the different fracture site distribution for the younger age groups with CP reflects the effect of CP or age. A prior study using private insurance claims data reported a higher 1-year risk of fractures in adults ≥18 years old with vs. without CP, but for the cohort without CP aged 18–64 years, ~70–85 % of the fractures occurred at the lower and upper extremities, with minimal contribution by the vertebral column and even less by the hip; however, in the elderly years, the fracture site distribution favored more hip and vertebral column sites (Whitney et al., 2021a). Although, comparison between studies is challenging due to differences in methodology, such as follow-up time and how fracture sites were grouped. Nevertheless, the different fracture site distribution in adults with CP likely reflects the greater extent of bone fragility in the lower vs. upper extremities, which may stem from lower mechanical loading leading to preferential deficits of bone structure and strength across the individual's body (Zhang et al., 2020; Al Wren et al., 2011; Chen et al., 2012; Jensen et al., 2020).

Knowledge of the site distribution of initial and subsequent fragility fractures has clinical implications. Certain fracture sites can be challenging to clinically manage, such as the distal femur, which is associated with heightened mortality rates compared to other fracture sites among the general elderly population (Mubark et al., 2020). The differential risks by fracture site may be exacerbated in adults with CP. For example, the absolute rate of incident cardiorespiratory disease and all-cause mortality has been shown to be higher for fragility fractures at the vertebral column and hip as compared to other lower extremity sites for adults with CP (e.g., 1-year mortality rate per 100 person-years: 9.0, 10.3, and 4.4, respectively) (Etter et al., 2020; Whitney et al., 2020a; Whitney et al., 2020b; Whitney et al., 2022a). However, the relative rate in these outcomes comparing adults with vs. without CP was higher for fragility fractures of the lower extremities as compared to the vertebral column and hip (e.g., 1-year mortality adjusted hazard ratio [95 % CI]: 1.95 [1.28–2.97], 1.39 [1.02–1.89], and 1.21 [0.88–1.66], respectively).

The limitations of this study that can directly influence interpretations must be discussed. First, claims data do not contain information about the severity of CP. It is possible that adults with more severe forms of CP who have more severe bone fragility (Whitney et al., 2022c) are disproportionately contributing to the higher fracture rates in the young adult years. However, this is likely less so for middle-aged and elderly groups because survival to older ages is more limited for those with more severe forms of CP (Blair et al., 2019). This may help to explain the lesser relative difference in outcomes with older age groups when compared to the same sex elderly without CP group. While bone fragility is worse for more vs. less severe forms of CP, it is still a problem for those with mild forms of CP (Whitney et al., 2017; Modlesky et al., 2009; Whitney et al., 2018a). Therefore, the estimates should be interpreted as a population-based effect averaging across levels of CP severity, where estimates may be slightly higher and slightly lower for more and less severe forms of CP, respectively, for the young adult age groups. Second, the representation and generalizability of study findings from this Medicare cohort with CP to the adult population with CP in the U.S. and internationally are not exactly known. However, as Medicare covers health insurance for many adults with CP with or without dual eligibility with Medicaid, it can be speculated that Medicare provides a reasonable representation of adults with CP in the U.S. Third, subsequent fracture risk may be overestimated in this study more for adults with vs. without CP. If there are delays in fracture healing or other complications that require an extended period for a healthcare visit that is billed for the same fracture event, the same fracture may have been designated as a distinct subsequent fracture. To mitigate this possible bias, this study required ≥6-months between the last claim for the preceding fracture and the first claim of the subsequent fracture if at the same site, which is a more conservative approach than what has been used previously (e.g., ≥3-month gap) (Keshishian et al., 2017; Liu et al., 2018). On the contrary, for some, subsequent fracture risk may have been underestimated as sidedness of the fracture site is not consistently available in the claims. If an individual sustained a fracture at the same site as the preceding fracture within 6-months, but on the contralateral limb, this subsequent fracture would have been disregarded. Fourth, this study was focused on generating novel epidemiologic evidence on fragility fracture risk, and did not examine risk factors other than age and sex (e.g., baseline comorbidities, medication use, race/ethnicity) or how the fractures were clinically managed (e.g., surgeries). This study helps to lay the foundation for such research. Finally, to examine longitudinal outcomes, this study identified individuals from 2008 to 2013, with the start of follow-up time beginning in 2009–2014 (to allow for a 1-year baseline period). This covers a long period in which the standard of care for bone fragility and CP may have changed. While the sensitivity analysis suggests no strong evidence that period bias largely impacts conclusions drawn, the majority of the cohorts started their follow-up in 2009 (>80 % for each cohort). Thus, future studies should consider more recent time periods to examine fracture or fracture-related outcomes.

Study findings have implications for clinicians who screen for and manage osteoporosis in patients with CP. In the absence of a fragility fracture, the diagnosis of osteoporosis is made using DXA scans. In young persons, the DXA reports a *Z*-score, the standard deviation of the mean using age, sex, and ethnicity-matched population. In the U.S., the reference standard is the National Health and Nutrition Examination Survey III database. A limitation of using this database is that the subjects mostly had normal bone development during growth, which does not apply to people with CP. Children with CP often develop small bones, even for a shorter stature and lower body mass, leading to small bones throughout the adult lifespan (Whitney et al., 2017; Modlesky et al., 2009; Whitney et al., 2022c; Noble et al., 2014). This poses a problem for clinical detection of osteoporosis as bone size alters DXA assessment of bone density such that fracture risk may be underestimated (Whitney et al., 2022c).

Clinical challenges also exist in identifying effective treatment strategies for osteoporosis for adults with CP, as this is an understudied topic for this population (Whitney et al., 2021b). Clinical trials have shown efficacy and safety of various osteoporosis medications, but these studies often include relatively healthy post-menopausal White women and often exclude individuals with CP and other forms of pediatric-onset disabilities (Bliuc et al., 2009; Crandall et al., 2014; Viswanathan et al., 2018; Fink et al., 2019; Cosman et al., 2018; Cosman et al., 2016; Lewiecki et al., 2019; Cummings et al., 1998; Delmas et al., 2002; Reid et al., 2018; Bliuc et al., 2019a; Bliuc et al., 2019b; van Geel et al., 2018; Kim et al., 2020). This calls into question if osteoporosis medications developed for/from the general population can be used safely and effectively for adults with CP. Given the earlier onset bone fragility, adults with CP may require fracture prevention strategies at a younger age and for a longer period of time compared to the general population. Adults with CP also have early onset morbidities and are exposed to a high number of medications (Whitney et al., 2018b; Whitney et al., 2021c; Whitney et al., 2020c), which may complicate early and long-term pharmaceutical treatment decision making given the unknown risks and benefits of various osteoporosis medications in the context of this heterogenous medical complexity.

## 5 Conclusion

This study provides novel epidemiologic evidence of the 5-year rate of fragility fracture and subsequent fragility fractures among women and men with CP across the adult lifespan. Findings suggest that adults with CP have a higher number of fragility fractures within a 5-year period as compared to elderly without CP, young adults <35 years old with CP have similar fragility fracture rates as the youngest-old (65–74 years old) without CP, and that subsequent fragility fracture rates are similar between young men and middle-aged women with CP as compared to elderly without CP. These findings emphasize the need for fracture prevention efforts to be implemented at a much younger age for adults with CP, possibly by ~5 decades younger. However, research is needed to develop fracture prevention and treatment strategies specific to this skeletally and medically complex population. Given the higher number of fragility fractures sustained over the 5-year period for adults with CP observed in this study, the intensity and composition of prevention and treatment efforts may need to be time-varying to match the skeletal needs at a given time.

## CRediT authorship contribution statement

**Daniel Whitney:** Conceptualization, Methodology, Sequestered Data, Formal analysis, Investigation, Resources, Writing- Original Draft, Writing- Review & Editing, Visualization, Project Administration, Funding acquisition, Data and Clinical Interpretation. **Gregory Clines:** Writing- Review & Editing, Data and Clinical Interpretation. **Aleda Leis:** Writing- Review & Editing, Interpretation, Validation. **Michelle Caird:** Writing- Review & Editing, Conceptualization, Data and Clinical Interpretation **Edward Hurvitz:** Writing- Review & Editing, Conceptualization, Data and Clinical Interpretation.

## Declaration of competing interest

None.
